# Impact of breast cancer characteristics on the development and time to liver metastases: population-based study

**DOI:** 10.1093/bjsopen/zrag036

**Published:** 2026-05-12

**Authors:** Marcus Sundén, Elin Lindqvist, Ellen Wahlqvist, Ellen Hansson, Charlotta Wadsten, Anne Andersson, Malin Sund, Oskar Hemmingsson

**Affiliations:** Department of Diagnostics and Intervention, Surgery, Umeå University, Umeå, Sweden; Department of Diagnostics and Intervention, Surgery, Umeå University, Umeå, Sweden; Department of Diagnostics and Intervention, Surgery, Umeå University, Umeå, Sweden; Department of Diagnostics and Intervention, Surgery, Umeå University, Umeå, Sweden; Department of Diagnostics and Intervention, Surgery, Umeå University, Umeå, Sweden; Department of Diagnostics and Intervention, Oncology, Umeå University, Umeå, Sweden; Department of Diagnostics and Intervention, Surgery, Umeå University, Umeå, Sweden; Department of Surgery, University of Helsinki and Helsinki University Hospital, Helsinki, Finland; Department of Diagnostics and Intervention, Surgery, Umeå University, Umeå, Sweden

**Keywords:** Neoplasm, human epidermal growth factor receptor 2 (HER2), oestrogen, progesterone, surrogate subtype, incidence

## Abstract

**Background:**

Breast cancer (Bc) is the leading cause of cancer-related death in women. In many patients, BC liver metastases (BCLM) are associated with short survival. The aims of this study were to investigate the risk of and time to BCLM in each BC surrogate subtype, and to determine the incidence of BCLM in a population-based setting.

**Methods:**

The Swedish national breast cancer registry identified patients with Bc in a regional cohort from 2009 to 2018. The cohort was followed until January 2023. Cox regression analysis was used to determine the risk of BCLM for each subtype. Kaplan–Meier estimates determined the probability of BCLM for each subtype over time.

**Results:**

In all, 7292 patients with Bc were included in the study. Distant metastases developed in 755 patients (10.4%); of these, 345 (45.7%) developed BCLM. The BCLM incidence rate was 8 per 1000 person-years. Only 13 patients had oligometastases isolated to the liver. Triple-negative, non-luminal human epidermal growth factor receptor 2 (HER2)-positive and luminal B cancers had the highest risk of BCLM. T category, nodal status, and Nottingham histological grade III were also strongly associated with BCLM. The median time from Bc diagnosis to BCLM was 36 months. Patients with HER2-positive BC subtypes developed BCLM early, at a median of only 9 months.

**Conclusion:**

Bc subtype is correlated to the risk and timing of BCLM development. BCLM are common in advanced Bc, but isolated oligometastases are rare.

## Teaser

The intrinsic breast cancer subtype is correlated with the risk of and time to the development of liver metastasis. Breast cancer liver metastases are common in advanced breast cancer, but isolated oligometastases are rare.

## Introduction

Despite advances in the treatment of disseminated breast cancer (BC), prognosis remains dismal. In a recent study investigating survival for patients with metastatic BC in Sweden, Valachis *et al.*^1^ reported a median survival of 30 months. There are large differences in survival depending on the BC surrogate subtype, histological grade, hormone receptor expression, and site of metastasis. Patients with bone metastases may survive for many years with stable disease, whereas cancer spread to visceral organs such as the liver is associated with poor prognosis^1–3^. It is also known from previous studies that the hormone receptor expression profile, specifically expression of oestrogen receptor (ER), progesterone receptor (PgR), and human epidermal growth factor receptor 2 (HER2), affects the establishment of metastases in specific organs^4–8^.

To optimize patient information, surveillance, and treatment protocols, it is important to study organ-specific metastases in subcohorts of BC. BCs are divided into surrogate subtypes according to the St. Gallen classification^9^, which is based on hormone receptor expression, histological grade, and cell proliferation rate. There are five subtypes in the St. Gallen classification^9^: luminal A, luminal B, luminal HER2-positive, non-luminal HER2-positive, and triple-negative BC (TNBC). Luminal A cancers have the best prognosis and have an affinity for bone in the metastatic setting. TNBC has the worst prognosis and is more prone to metastasize to the brain or viscera.

Local treatment, such as surgery, ablation, or stereotactic radiation therapy, may prolong survival in select patients with BC liver metastases (BCLM). In this scenario, surveillance and early detection become increasingly important. Evidence for the treatment of local metastases comes from case series and case-control studies^10^, but there have been no randomized prospective trials on local treatment of BCLM. In most studies, local treatment for BCLM is limited to patients with so-called oligometastases (< 5 BCLM)^11^. To design trials on metastatic disease, it is important to determine the incidence and time to organ-specific metastases. This is also true for recommendations on surveillance of BC recurrence. Patients with the highest risk of treatable recurrence should have the most rigorous follow-up. In current guidelines, there are no recommendations for organ-specific imaging-guided follow-up^10,12^.

Although studies on BC recurrence may suffer from inclusion bias and loss of follow-up data, the present study used a population-based registry to determine BCLM incidence, risk factors, and the timing of metastases. Swedish patients with BC are registered in the National Breast Cancer Register (NBCR)^13^. The NBCR is validated and is linked to the National Cancer Register and the Total Population Register. The inclusion rate is nearly 100% at the time of primary Bc.

The aim of this study was to improve BC treatment by increasing knowledge of BCLM. Specifically, the study sought to determine risk factors for BCLM with particular emphasis on BC subtypes, the incidence of BCLM, and the time to BCLM.

## Methods

All patients diagnosed with BC between 2009 and 2018 in the four northernmost counties in Sweden (Norrbotten, Västerbotten, Jämtland, Västernorrland) were identified in the NBCR.

Data were retrieved from the NBCR, including the year of and age at diagnosis of BC, sex, vital status (dead or alive), date of death, clinical tumour node metastasis (TNM) staging system classification, receptor (ER, PgR, HER2) status, proliferation marker Ki-67, Nottingham histological grade (NHG), locoregional and distant metastases, and adjuvant treatment. From the registry data, primary tumours were classified into surrogate subtypes according to the St. Gallen classification^9^. BCs were considered ER and PgR positive when the expression of these receptors was > 10%. Tumours were considered HER2 positive if they had a score of 3 + on immunohistochemical analysis or were positive on silver or fluorescence *in situ* hybridization. The proliferation index (Ki-67) was classified as low, intermediate, or high in the NBCR. If the Ki-67 category was not available, Ki-67 expression < 6% was considered low, 6–29% was considered intermediate and > 30% was considered high. Tumours were categorized as NHG I, II, or III. Luminal (ER-positive) tumours were classified as Luminal A if they were NHG I and luminal B if they were NHG III. If the NHG was II or not available, tumours were registered as Luminal A or B if Ki-67 was low or high, respectively. Luminal tumours with NHG II and an intermediate proliferation index (Ki-67) were classified as luminal B if PgR expression was < 20%. Tumours were classified as TNBC when they were negative for ER, PgR, and HER2.

The NBCR lacks sufficient data on recurrent cancer and metastases. Therefore, this information was retrieved from digital patient records at the departments of surgery in each region and radiology reports, and was collated in case report forms. The radiology module in the regions contains all examinations performed in public hospitals. Data on metastases (site, numbers, and size) were registered. All patients with metastases (both synchronous and metachronous) were included in the cohort. Among patients with advanced disease, those with BCLM as the primary metastasis site and those who later developed BCLM were included. Oligometastasis was defined as five or fewer metastases.

The most recent contact with a department of surgery, participation in the mammography screening programme, or any other imaging examinations were used to analyse recurrence. The latest entry in the patients records was made 27 January 2023.

Patients with bilateral BC and/or patients with recurrent BC were excluded from the study because it was not possible to determine which tumour metastasized.

### Statistical analysis

Descriptive statistics are used to describe baseline characteristics. The significance of differences between groups was analysed using χ^2^ tests. A two-tailed *P* value < 0.05 was considered significant.

Cox regression analysis was used to determine hazard ratios (HRs) for different risk factors for BCLM and oligo-BCLM. Kaplan–Meier plots were used to investigate the time to BCLM for BCs according to subtype and receptor expression.

This study was approved by the Swedish Ethical Review Authority (Dnr 2020-02165; holder, Oskar Hemmingsson). After the initial data collection, all data were pseudonymized to minimize the impact on patient privacy.

## Results

### Characteristics of the study cohort

From the NBCR, 7446 patients with a BC diagnosis between 2009 and 2018 in Northern Sweden were included. Of these patients, 154 were excluded due to bilateral or multiple BCs and loss to follow-up. The final study cohort consisted of 7292 patients, who were followed up until the latest contact (as described above) or time of death, whichever came first. The cohort is named the North Sweden Breast Cancer Cohort (NSBCC). The median follow-up time was 64 months.

Most of the cohort were women (7248/7292, 99.4%). The mean age at BC diagnosis was 64, ranging from 19 to 101 years (*Table 1*). Approximately two-thirds of patients (64%) underwent breast-conserving surgery and one-third (35%) underwent total mastectomy. Most patients had ductal BC (4880/6251, 78.1%). The most common surrogate subtype in the cohort was luminal A (2094/4342, 48.2%) followed by luminal B (1054/4342, 24.3%). Almost 90% (5370/6064) of patients had ER-positive Bc. Adjuvant chemotherapy was administered to 32.3% of patients, and in most cases was anthracycline based. Adjuvant radiation therapy was administrated to 74.1% of patients, 61.4% received endocrine therapy, and 10.1% received targeted anti-HER2 therapy (mostly trastuzumab).

**Table 1:** Baseline data

	All patients	Metastatic disease	BCLM	Oligo-BCLM
Cohort (*n*)	7292	755	345	84
**Sex**				
Female	7248 (99.4%)	749 (99.2%)	341 (98.8%)	83 (98.8%)
Male	44 (0.6%)	6 (0.8%)	4 (1.2%)	1 (1.2%)
**Mean Age at BC (full range)**	64 (19–101)	65 (24–95)	63 (24–90)	63 (24–88)
**ER Bc**				
Positive	5370 (88.6%)	478 (79.0%)	225 (79.2%)	54 (79.4%)
Negative	692 (11.4%)	127 (21.0%)	59 (20.8%)	14 (20.6%)
Missing (*n*)	1228	150	61	16
**PgR Bc**				
Positive	4554 (79.4%)	374 (67.0%)	170 (65.9%)	33 (56.9%)
Negative	1182 (20.6%)	184 (33.0%)	88 (34.1%)	25 (43.1%)
Missing (*n*)	1556	197	87	26
**HER2 Bc**				
Positive	862 (18.6%)	123 (25.3%)	55 (23.0%)	15 (29.4%)
Negative	3781 (81.4%)	363 (74.7%)	184 (77.0%)	36 (70.6%)
Missing (*n*)	2649	269	106	33
**Ki-67 Bc**				
Low	2688 (43.1%)	172 (25.6%)	72 (22.1%)	19 (23.8%)
Intermediate	924 (14.8%)	95 (14.2%)	33 (10.1%)	9 (11.3%)
High	2622 (42.1%)	404 (60.2%)	221 (67.8%)	52 (65.0%)
Missing (*n*)	1058	84	19	4
**NHG Bc**				
I	1187 (17.8%)	43 (7.7%)	9 (4.1%)	3 (4.3%)
II	2973 (44.6%)	195 (34.8%)	75 (34.1%)	22 (31.9%)
III	2125 (31.9%)	272 (48.6%)	136 (61.8%)	38 (55.1%)
Missing (*n*)	1007	245	125	21
**Surrogate subtype Bc**				
Luminal A	2094 (48.2%)	111 (25.5%)	42 (20.3%)	8 (19.5%)
Luminal B	1054 (24.3%)	140 (32.1%)	78 (37.7%)	11 (26.8%)
Luminal HER2 positive	549 (12.6%)	67 (15.4%)	30 (14.5%)	8 (19.5%)
Non-luminal HER2 positive	207 (4.8%)	38 (8.7%)	16 (7.7%)	2 (4.9%)
TNBC	438 (10.1%)	80 (18.3%)	41 (19.8%)	12 (29.3%)
Missing (*n*)	2950	319	138	43
**Histological type of Bc**				
Ductal	4880 (78.1%)	505 (78.1%)	258 (84.9%)	63 (85.1%)
Lobular	832 (13.3%)	99 (15.3%)	34 (11.2%)	7 (9.5%)
Other	539 (8.6%)	43 (6.6%)	12 (3.9%)	4 (5.4%)
Missing (*n*)	1041	108	41	10
**Size of Bc**				
< 20 mm	3376 (59.0%)	283 (38.0%)	109 (31.9%)	29 (35.4%)
> 20 mm	2344 (41.0%)	462 (62.0%)	233 (68.1%)	53 (64.6%)
Missing (*n*)	1572	10	3	2
**Axillary metastases Bc**				
Positive	961 (13.3%)	291 (39.6%)	151 (45.1%)	40 (48.2%)
Negative	6258 (86.7%)	444 (60.4%)	184 (54.9%)	43 (51.8%)
Missing (*n*)	73	20	10	1

Values are *n* (%) unless otherwise stated. Percentages refer to valid cases only. BCLM, breast cancer liver metastasis; BC, primary breast cancer; ER, oestrogen receptor; PgR, progesterone receptor; HER2, human epidermal growth factor receptor 2; NHG, Nottingham histological grade; TNBC, triple-negative breast cancer.

At the time of BC diagnosis, 961 patients (13.3%) had axillary lymph node metastases (clinical staging).

Only three patients in this study had local treatment (resection, ablation or stereotactic radiation therapy) for BCLM.

### Incidence of BCLM

During the study period, 755 patients (10.4%) developed distant metastases. Among all patients, 345 (4.7%) developed BCLM during follow-up. Hence, among patients with metastases, almost half (45.7%) had BCLM. Of all distant recurrences, 125 (16.6%) were isolated BCLM with no other metastases initially.

Eighty-four patients (24.3% of all patients with BCLM) were diagnosed with oligo-BCLM (< 5 BCLM). Only 13 patients (3.8% of all patients with BCLM and 10.4% among those with isolated BCLM) had isolated oligometastases.

For the total population of approximately 900 000 inhabitants in the four counties, there were, on average, four new cases of BCLM per year and per 100 000 inhabitants during the study period.

The BCLM incidence rate in the study cohort, expressed as the number of cases in relation to follow-up time, was 8 per 1000 person-years. The corresponding number for oligo-BCLM was 2 per 1000 person-years.

### Risk of BCLM according to surrogate subtype and tumour characteristics

As expected, node-positive BC is more common in patients with metastases, and the distribution of BC subtypes differed significantly in the group of patients who developed metastases compared with the entire study cohort. There was a much higher rate of TNBC among patients with metastases (*Table 1*).

In a Cox regression analysis of risk for BCLM, there was a significant difference in risk for the different surrogate subtypes (*Table 2*). Patients with luminal A cancers were least likely to be diagnosed with BCLM. TNBC carried the greatest risk for BCLM, approximately fivefold greater than for luminal A type BC (HR 5.2; 95% confidence interval (c.i.) 3.4 to 8.0; *P* < 0.001). A high histological grade and/or a high Ki-67, primary tumour size > 20 mm, and lymph node metastases at diagnosis significantly increased the risk of BCLM (*Table 2*). However, age and histological tumour type (ductal or lobular) did not affect the risk of BCLM.

**Table 2:** Cox regression analysis of risk factors for BC liver metastasis

	Hazard ratio*	*P*
**Age**		
> 60 years	Reference	
< 60 years	1.08 (0.86, 1.34)	0.514
**Surrogate subtype Bc**		
Luminal A	Reference	
Luminal B	3.95 (2.69, 5.80)	< 0.001
Luminal HER2 positive	3.00 (1.89, 4.81)	< 0.001
Non-luminal HER2 positive	4.48 (2.51, 8.00)	< 0.001
TNBC	5.02 (3.22, 7.83)	< 0.001
**Receptor**		
ER positive	0.49 (0.37, 0.66)	< 0.001
PgR positive	0.50 (0.39, 0.66)	< 0.001
HER2 positive	1.46 (1.07, 1.97)	0.015
**Ki-67 Bc**		
Low	Reference	
Intermediate	1.26 (0.82, 1.94)	0.298
High	3.54 (2.70, 4.65)	< 0.001
**NHG Bc**		
I	Reference	
II	3.27 (1.64, 6.53)	< 0.001
III	8.37 (4.26, 16.45)	< 0.001
**Histological type of Bc**		
Ductal	Reference	
Lobular	0.78 (0.54, 1.12)	0.176
Other	0.49 (0.26, 0.92)	0.025
**Size of Bc**		
< 20 mm	Reference	
> 20 mm	5.70 (4.52, 7.19)	< 0.001
**Axillary metastases Bc**	6.52 (5.22, 8.13)	< 0.001

*Values in parentheses are 95% confidence intervals. Bc, primary breast cancer; HER2, human epidermal growth factor receptor 2; TNBC, triple-negative breast cancer; ER, oestrogen receptor; PgR, progesterone receptor; NHG, Nottingham histological grade.

A similar analysis investigating the risk of developing oligo-BCLM showed that, although not significant, non-luminal HER2-positive BC carries the greatest risk compared with luminal A BC (HR 4.8; 95% c.i. 0.9 to 25.5; *P* = 0.071). Patients with TNBC also had a high risk of developing oligo-BCLM (HR 4.0; 95% c.i. 1.5 to 10.8; *P* = 0.007).

The risk of BCLM was lower for patients with ER- or PgR-positive BC (HR 0.5 (95% c.i. 0.4 to 0.7; *P* < 0.001) for both; *Table 2*). Amplification of the *HER2* gene increased the risk of BCLM (HR 1.5; 95% c.i. 1.1 to 2.0; *P* = 0.015; *Table 2*).

### Time to BCLM

**Fig. 1:**
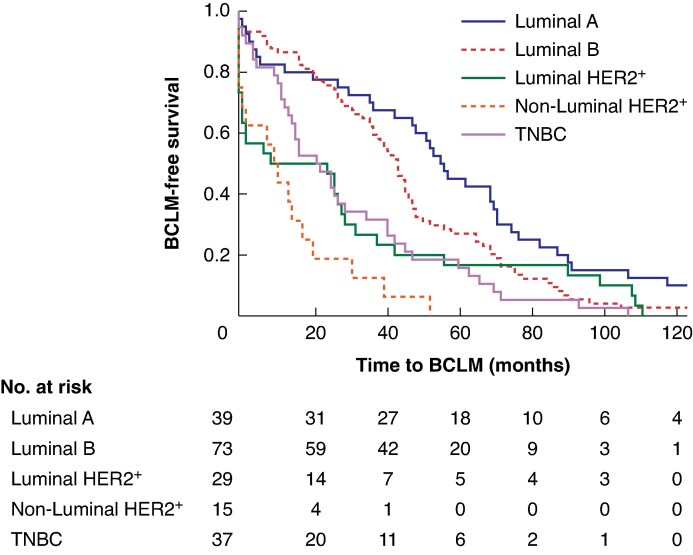
Time to BCLM for each surrogate subtype Only patients who developed BCLM are included in the analysis. BCLM, breast cancer liver metastasis; HER2^+^, human epidermal growth factor receptor 2 positive; TNBC, triple-negative breast cancer.

The median time from breast cancer to BCLM was 36 months for all surrogate subtypes. The timing of metastases differed significantly between subtypes (*Fig. 1*). The two HER2-positive subtypes, luminal HER2-positive and non-luminal HER2-positive BC, had the shortest time to BCLM (only 9 and 10 months, respectively). The median time to BCLM for TNBC was 22 months, whereas luminal A cancer had the longest time to BCLM (57 months).

**Fig. 2:**
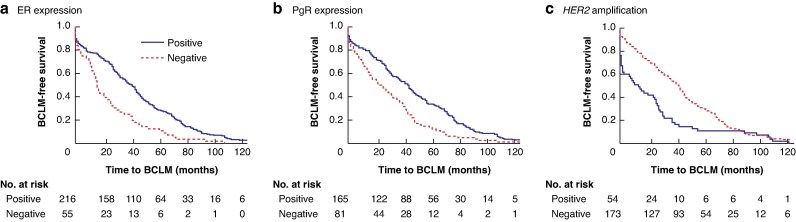
Time to BCLM in relation to receptor expression **a** ER expression, **b** PgR expression, and **c** amplification of the *HER2* gene. There were significant differences in time to BCLM between patients positive and negative for each receptor subtype and *HER2* gene amplification (*P* < 0.001 for all). Note, only patients who developed BCLM are included in the analysis. BCLM, breast cancer liver metastasis; ER, oestrogen receptor; PgR, progesterone receptor; HER2, human epidermal growth factor receptor 2.

Analysing the time to BCLM according to individual receptor status showed that ER- and PgR-positive tumours had a similar median time to BCLM (42 months and 44 months, respectively). For HER2-positive BC, the time to BCLM was much shorter, at only 14 months. For all receptors, the difference between positive and negative groups was highly significant (*Fig. 2*): ER- and PgR-negative tumours progressed more rapidly to BCLM, and the same was true for HER2-positive tumours.

## Discussion

Metastatic BC continues to be a serious diagnosis. Knowledge about cancer cell affinity for various metastatic sites may be of great importance for tailoring surveillance programmes and treatment protocols for individual patients. Liver metastasis is a leading cause of death in metastatic BC, and local treatment of BCLM is an option that may prolong survival in select patients^14–17^. The aim of the present study was to investigate which patients are at the greatest risk of developing BCLM. Earlier studies were based on the sampling of a population and estimations of risk^4,5,8^. A strength of the present study is that it was performed on a population-based cohort in a large geographically confined area of Sweden. The patient cohort was identified in an externally validated national register with an inclusion rate near 100%. Most patients with BC in Sweden are treated within the public healthcare system. BC treatment follows national guidelines and resources for investigation and treatment were equal in the four regions^13^. According to the Swedish guidelines, imaging to screen for metastases is based on risk evaluation and clinical symptoms, and is not routinely performed at the time of BC diagnosis.^13^ The same is true for the detection of metachronous metastases. No organ-specific imaging-guided follow-up is recommended in guidelines.^13^

Only a small number of patients were excluded in the present study, approximately 2% of the NBCR cohort. The size of the cohort, just above 7000 patients, made it possible to demonstrate significant differences between groups of patients. In the present study, patients with luminal A tumours were, as expected^4,5,8^, least likely to develop BCLM. HER2-positive cancers and TNBC metastasized more often to the liver during the course of the disease than did other tumour subtypes. Patients with TNBC had the highest risk of BCLM. Other factors that increased the risk of BCLM were a high proliferation rate, high histological grade, large Bc size, and metastases to regional lymph nodes at diagnosis. Similar results were found by Wu *et al.*^5^ in a large registry-based study, with HER2-positive tumours more likely to metastasize to the liver.

In the present study, the timing of BCLM differed significantly between different surrogate subtypes. Patients with luminal A-type cancers developed BCLM later than patients with other subtypes. The relatively long follow-up time (median 64 months) is a strength of the present study and covers most metastases from aggressive BC subtypes. Still, metastases from luminal tumours can arise after more than 10 years (*Fig. 1*), leading to a possible underestimation of the incidence of BCLM.

HER2 expression in the primary BC increases the risk of BCLM, and BCLM develops early in patients with HER2-positive BC. With existing efficient HER2-targeted treatments, a consequence of these results could be that patients with HER2-positive BC should be offered imaging-based surveillance to detect liver recurrences, enabling early systemic treatment and possibly local treatment.

Approximately 5% of all patients in the present study developed BCLM and approximately one-quarter within the BCLM group had oligometastases. Many of these patients had synchronous metastases in other sites. This means that patients eligible for local treatment for BCLM are a highly select group. Nevertheless, for these patients, local treatment may improve survival. In many centres stable bone metastasis is not a contraindication to surgery or other local treatment, indicating that more patients can benefit from local treatment.

In the present cohort, only three patients had local treatment for BCLM. The authors have previously reported that approximately five patients per year have surgery for BCLM in Sweden^14^. The reason for this may be the lack of liver-focused surveillance and clear guidelines on how to select patients for local treatment. In the present study, isolated oligometastases were very rare, detected in only 13 patients in this large cohort.

A weakness of this study is insufficient information about oncological treatment at the time of BCLM. Another weakness is that imaging reports do not always describe in detail all findings regarding the number and size of metastases. Further, it was not possible to classify all tumours into subtypes because necessary information about receptors and proliferation was missing in some pathological reports.

Recent advances in molecular profiling of BC tumour cells using DNA- and RNA-based methods may provide even more exact and early identification of groups of patients at high risk of BCLM. For example, the ability to detect circulating tumour DNA in the future may enable BCLM to be detected earlier.

Continuing efforts are needed to improve knowledge of BCLM, to improve follow-up in patients at high risk so as to detect BCLM early, and to provide the best systemic and local treatment available.

Primary BC surrogate subtype, T category, N category, histological grade, and proliferation determine the risk and timing of BCLM. *HER2*-amplified Bc and TNBC are associated with early BCLM. BCLM is common in advanced BC, but isolated oligometastases amenable to local treatment are rare.

## Data Availability

Data supporting this study are available from the corresponding author, but access to the data is subject to approval and a data transfer agreement due to legal and ethical reasons.
